# GM2-Gangliosidosis (Sandhoff and Tay Sachs disease): Diagnosis and Neuroimaging Findings (An Iranian Pediatric Case Series)

**Published:** 2014

**Authors:** Parvaneh KARIMZADEH, Narjes JAFARI, Habibeh NEJAD BIGLARI, Sayena JABBEH DARI, Farzad AHMAD ABADI, Mohammad-Reza ALAEE, Hamid NEMATI, Sasan SAKET, Seyed Hasan TONEKABONI, Mohammad-Mahdi TAGHDIRI, Mohammad GHOFRANI

**Affiliations:** 1 Pediatric Neurology Research Center, Shahid Beheshti University of Medical Sciences, Tehran, Iran; 2 Pediatric Neurology Center of Excellence, Department of Pediatric Neurology, Mofid Children Hospital, Faculty of Medicine, Shahid Beheshti University of Medical Sciences, Tehran, Iran; 3 Department of Pediatric Neurology, Ardebil University of Medical Sciences, Ardabil, Iran; 4 Department of Pediatric Endocrinology, Faculty of Medicine, Shahid Beheshti University of Medical Sciences, Tehran, Iran

**Keywords:** Sandhoff disease, Tay Sachs disease, Neurometabolic disorders, Genetic disorders

## Abstract

**Objective:**

GM2-Gangliosidosis disease is a rare autosomal recessive genetic disorder that includes two disorders (Tay–Sachs and Sandhoff disease).These disorders cause a progressive deterioration of nerve cells and inherited deficiency in creating hexosaminidases A, B, and AB.

**Materials & Methods:**

Patients who were diagnosed withGM2-Gangliosidosis in the Neurology Department of Mofid Children’s Hospital in Tehran, Iran from October 2009 to February 2014were included in our study. The disorder was confirmed by neurometabolic and enzyme level detection of hexosaminidases A, B, and AB in reference to Wagnester Laboratory in Germany. We assessed age, gender, past medical history, developmental status, clinical manifestations, and neuroimaging findings of 9 patients with Sandhoff disease and 9 with Tay Sachs disease.

**Results:**

83% of our patients were the offspring of consanguineous marriages. All of them had a developmental disorder as a chief complaint.

38%of patients had a history of developmental delay or regression and 22% had seizures. The patients with Sandhoff and Tay Sachs disease were followed for approximately 5 years and the follow-up showed all patients were bedridden or had expired due to refractory seizures, pneumonia aspiration, or swallowing disorders.

Neuro-imaging findings included bilateral thalamic involvement, brain atrophy, and hypo myelination in near half of our patients (48%).

**Conclusion:**

According to the results of this study, we suggest that cherry-red spots, hyperacusis, refractory seizures, and relative parents in children with developmental delay and/or regression should be considered for assessment of GM2-Gangliosidosis disease.

## Introduction

Sandhoff disease is a rare autosomal recessive metabolic disorder that has three clinical subtypes (infantile, juvenile, and adult forms)(1,2).The infantile form presents with progressive neurologic impairment, hyperacusis, hypotonia, and bilateral cherry-red spots in the macular region of the retina and seizures(3).The juvenile form manifests with dementia, cerebellar ataxia, mental retardation, and spinal muscular atrophy(4).The clinical manifestation of the adult form of Sandhoff disease varies widely from spinocerebellar degeneration to motor neuron disorders are often reported(5-8).

Tay-Sachs disease (TSD) is a neurodegenerative lysosomal disorder with an autosomal recessive inheritance caused by β-hexosaminidase α-subunit (HEXA) mutations (9).

The diagnosis of this disorder is based on hexosaminidases A, B, and AB level detection. 

Decreased levels of hexosaminidases A and B are seen in patients with Sandhoff but solitarily decreased levels of hexosaminidases A are seen in Tay Sachs disease. 

In this study, we present 5 years of experience about Sandhoff and Tay Sachs disorder from the Pediatric Neurology Research Center of Mofid Children’s Hospital, Tehran, Iran.

## Materials & Methods

A total of 18 cases affected byGM2-gangliosidosis disease were assessed in our study from October 2009 to February 2014 in the Neurology Department of Mofid Children’s Hospital, which is the referral center for neurometabolic diseases in Iran. 

The diagnosis was performed based on clinical manifestations, neuro-imaging findings, and, finally, laboratory assessment of decreased total hexosaminidase enzyme activity for Tay Sachs and Sandhoff disease from a metabolic laboratory in Germany. The data from patients were collected was age, gender, past medical history, developmental status, general appearance, and clinical neuro-imaging findings. 

Unfortunately, this disorder is incurable and treatment consists of anticonvulsants to manage seizures, proper nutrition, and rehabilitation. The children’s diet was carefully controlled. The data were analyzed by descriptive methods and no statistical testing was applied.

Institutional ethical approval for the conduct of this study was obtained from the Pediatric Neurology Research Center (Shahid Beheshti University of Medical Sciences). All parents signed a written consent for participation in this study.

## Results

In our study 18 patients with GM2-Gangliosidosis (9 patients with Sandhoff and 9 with Tay Sachs disease) were included. There were 10 males and 8 females with a mean age at time of presentation of15 months and an average age of18 months. Hospitalization history for 2 patients from maternal preeclampsia and for 4 patients from pneumonia (1 patient) and icter (3 patients). 

The first and chief complaint in 100% of the patients were neurological disorders, such as developmental delays (6 patients), developmental regression (5 patients), or both (7 patients); and 4 patients complained of simultaneous seizures.

During developmental assessment, 66% of patients showed developmental regression. The average age for developmental regression was 15 months and the mean age was 12 months(3 months before admission and detection time).Four patients had a history of recurrent hospitalization because of respiratory and urinary tract infections. Eight patients had central hypo tonicity (decreased tonicity and increased DTR) and5 patients had spasticity.55% of patients had visual disorders and fix-follow did not exist during physical examinations. 

Nine patients had a history of seizure with the most common form of seizure were tonic-myoclonic seizures.

**Fig.1:**
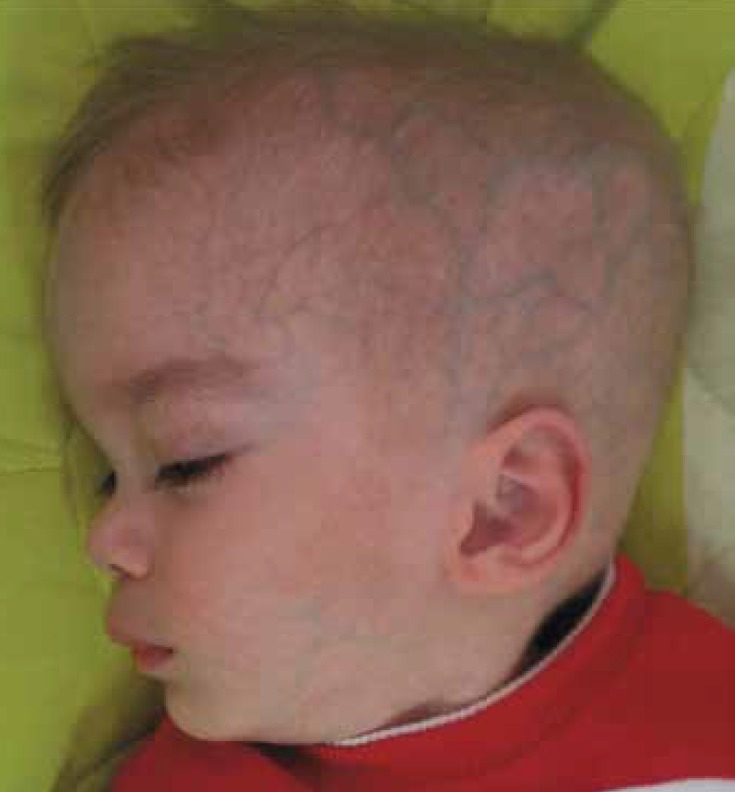
A 9 -month –boy patient of GM2-gangliosidosis with a dysmorphic face, protruding forehead, and a depressed nasal bridge

**Fig. 2:**
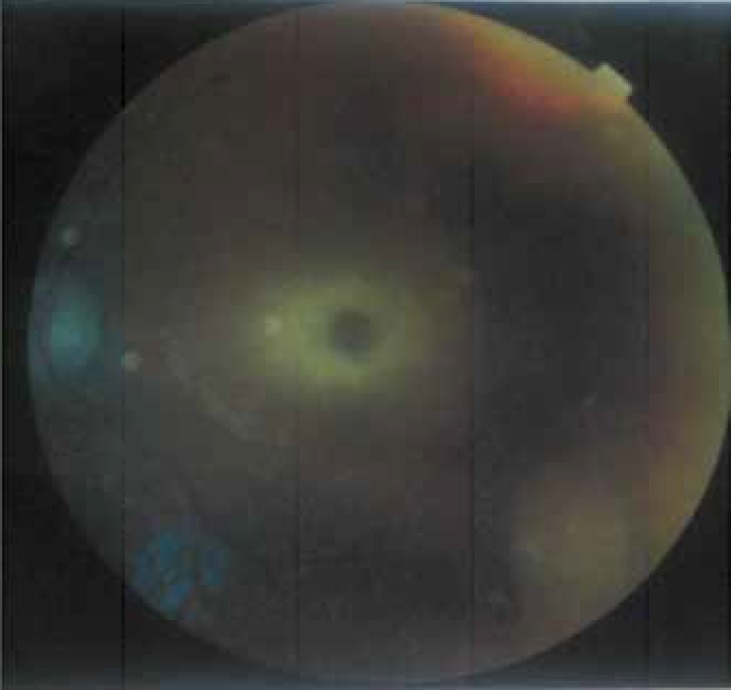
Ophtalmoscopic pattern of 9- month-old boy patient with GM2- gangliosidosis characterized with cherry-red spot in retinal Exam

Seven patients had hyperacusis. 55% of patients had a dysmorphic face with protruding forehead, depressed nasal bridge, and hypertelorism. Five patients had blond hair (Fig. 1). One patient had hepatomegaly and another had hepato splenomegaly. Weight in 6 patients was below the 3 percentile and height in 10% of patients was below the 5 percentile. Three patients had microcephaly, seven patients showed macrocephaly, and the remainder had normal head circumferences.83% of patients were the offspring from consanguineous marriages. Four patients had a family history of seizures and mental retardation. No abnormality was observed in other physical examinations (chest and abdomen).Cherry-red spots were seen in 88% of patients (Fig.2). In lab data, three patients had increased levels of AST and ALT. 

**Fig 3:**
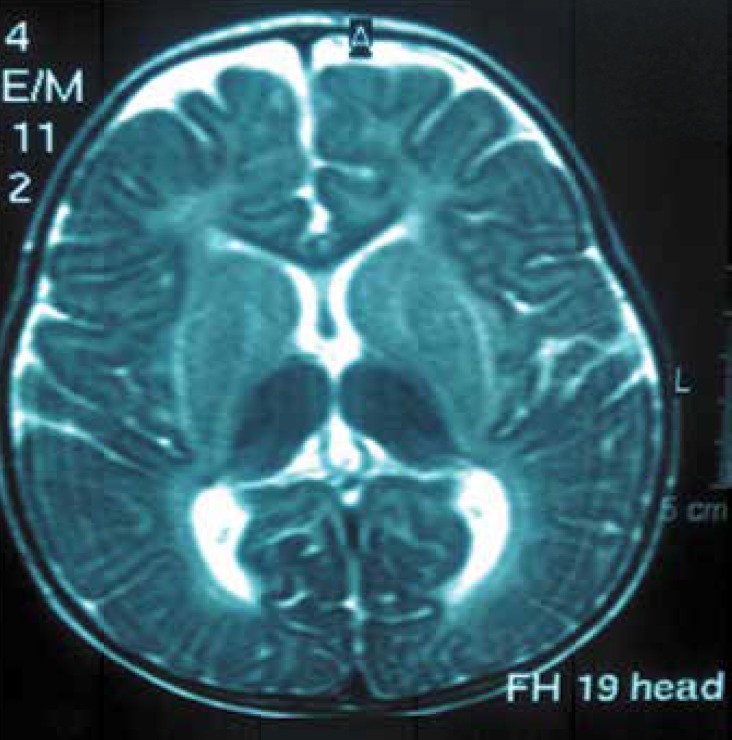
An 11-month-old female patient with GM2-gangliosidosis with bilateral thalamic involvement inT2 sequence of the brain MRI

**Fig 4:**
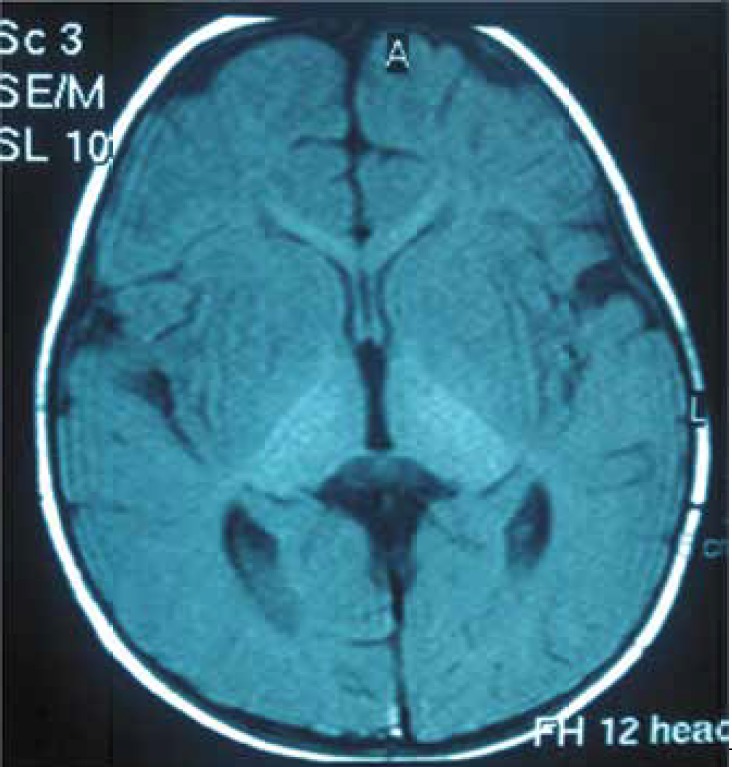
A 10-month-old male patient with GM2-gangliosidosis with bilateral thalamic involvement in brain imaging

The neuroimaging data showed that39% of patients had normal neuro-imaging; 22% of patients had bilateral thalamic involvement; 22% had brain atrophy, and 16% of patients showed demyelination delay. (Fig. 3 and 4). 

## Discussion

Sandhoff disease is the one type ofGM2-gangliosidosis may present with developmental regression within the first 6 months of life (10). Tay-Sachs diseaseis another type of GM2-gangliosidosis well-known inherited disease caused by an accumulation of gangliosidosis in the retina and brain (11).

Patients with GM2-Gangliosidosis (9 patients with Sandhoff disease and 9 patients with Tay Sachs disease) were referred to our tertiary center equally. There were no sexual differences. The main chief complaint from all patients for detection time was developmental delay or regression.

Seizures were indicated in some cases. Four patients had a history of neonatal hospitalization (icter, infection) and four patients had recurrent hospitalization due to respiratory distress and Urinary Tract Infections (UTI).

72% of patients had central hypotonicity or spasticity. 39% of patients had hyperacusis; 56% of patients had specific facial features;28% of patients had blonde hair and a sunken nose bridge; and 28% of patients had hypertelorism. 83% of patients were offspring of consanguineous marriages. Therefore, having relative parents is an important factor in GM2-gangliosidosis detection. 22% of our Sandhoff patients showed bilateral thalamic involvement as hyper-signal intensity of white matter in T2 weighted.

Yun YM et al reported MRIs that showed low signal intensity at the thalamus and high signal intensity at white matter of brain in T2-weighted(1). These manifestations in the infantile form of Sandhoff disease were caused by an accumulation of calcium due to intracellular storage of GM2-ganglioside(12-13).

Kokot W et al made proper diagnoses of Sandoff and Tay Sachs disease in their patients based on an early eye fundus examination and seeing cherry-red spots in the central area(14). 55% of our patients did not have fixfollow on visual examination and cherry-red spots were seen in 88% of patients.

The treatment of GM2-gangliosidosis is based on patient complaints such as management of the epileptic seizures and an intervention program for the neurological retardation. Case mortality in the infantile form of this disorder with severe neurological deterioration occurs before the age of 4 (3).

Organomegaly was seen in only 2 patients and was not an important segregation criterionto distinguishing Sandhoff from Tay Sachs disease in our study. Likewise, Barness et al. also did not report organomegaly in patients with Sandhoff disease (15). However, Arisoy et al. reported a case of Tay Sachs in a twelve-month-old female patient with macrocephaly, hyperacusis, cherry-red spots, and without organomegaly (16). Ozkara et al. showed that in 11 out of 18 infantile patients with Sandhoff disease, there was no evidence of organomegaly, while the remaining seven had mild organomegaly(17). They also reported that 21% of marriages in their patients were consanguineous (17).

Therefore, the lack of organomegaly or macrocephaly in the clinical manifestation of our studied group could not preclude a GM2-gangliosidosis diagnosis. 

Seven patients had macrocephaly, three patients had microcephaly, and eight patients had a normal head circumference. Therefore, macrocephaly, microcephaly, and normal head circumferences were not considered significant in our assessment. 

Our patients with GM2-gangliosidosis came to our special center and an exact evaluation was done. A high frequency of consanguineous marriage between the patients’ parents must be considered remarkable. In these autosomal recessive disorders, because there is no curative therapy, genetic counselling is important and necessary to prevent the burden of GM2-gangliosidosis as a neuro-metabolic disorder. 

**In conclusions, **the first and chief complaint in 100% of the patients were neurological disorders.

83% of patients were offspring of consanguineous marriages and cherry-red spots were seen in 88% of patients. Therefore, cherry-red spots, hyperacusis, refractory seizures, and relative parents in patients with developmental delay or regression are the most important factors in the diagnosis of suspected patients.

These factors must be considered as assessment for GM2-gangliosidosis disease.
